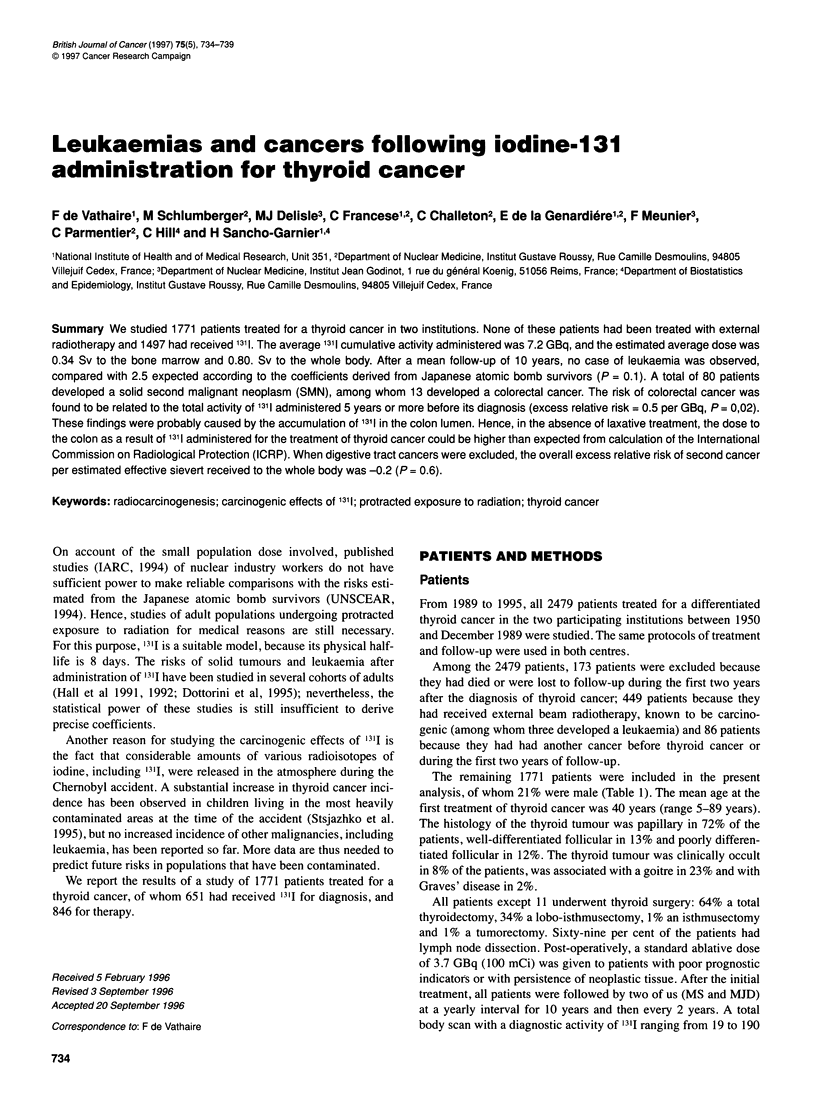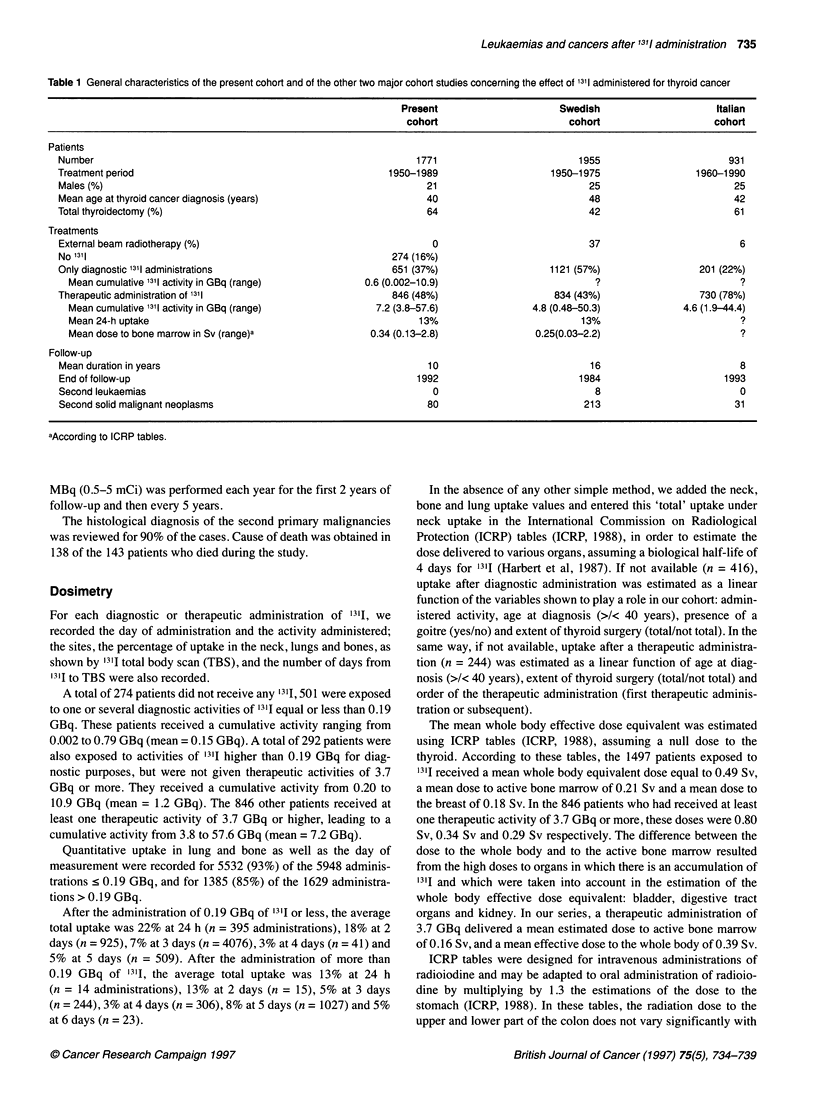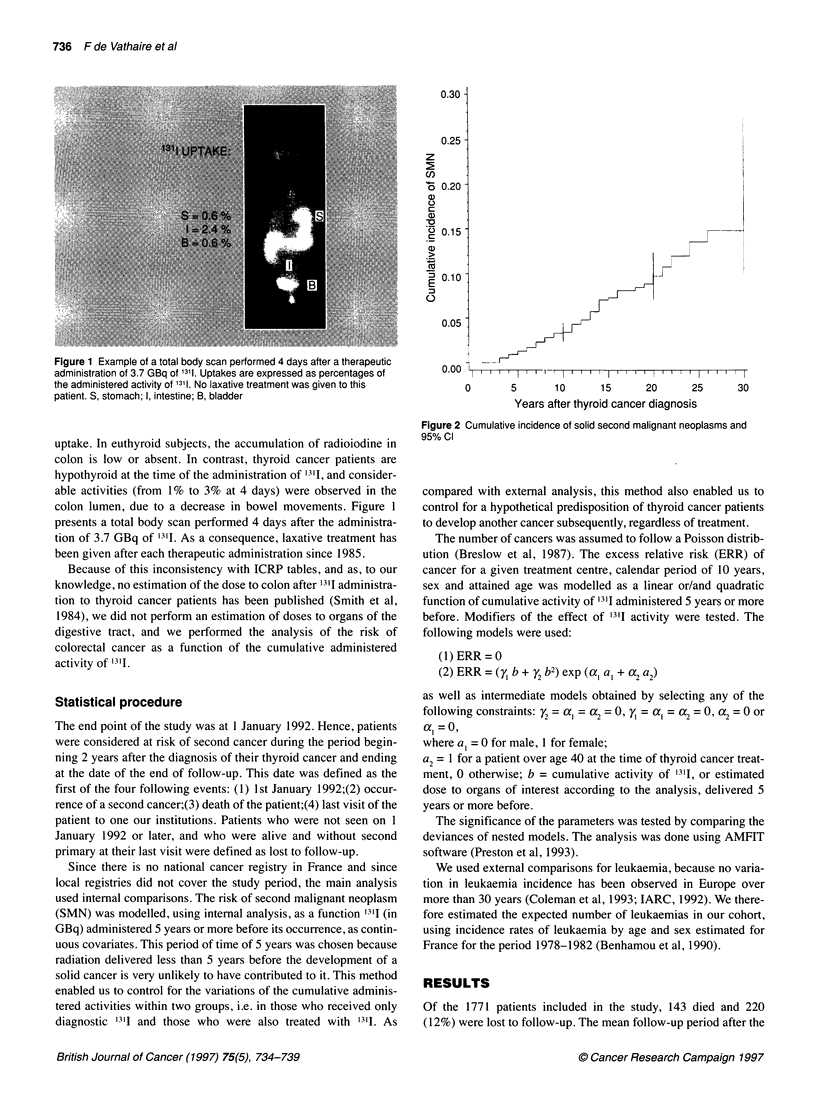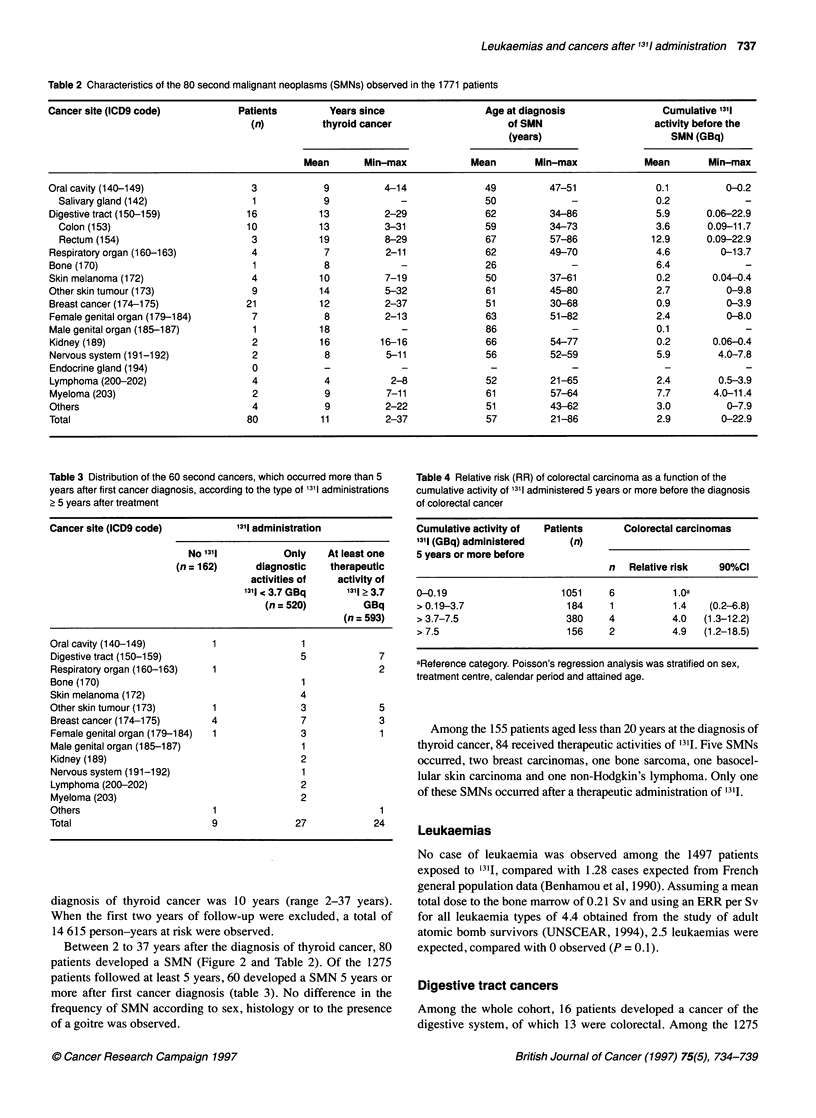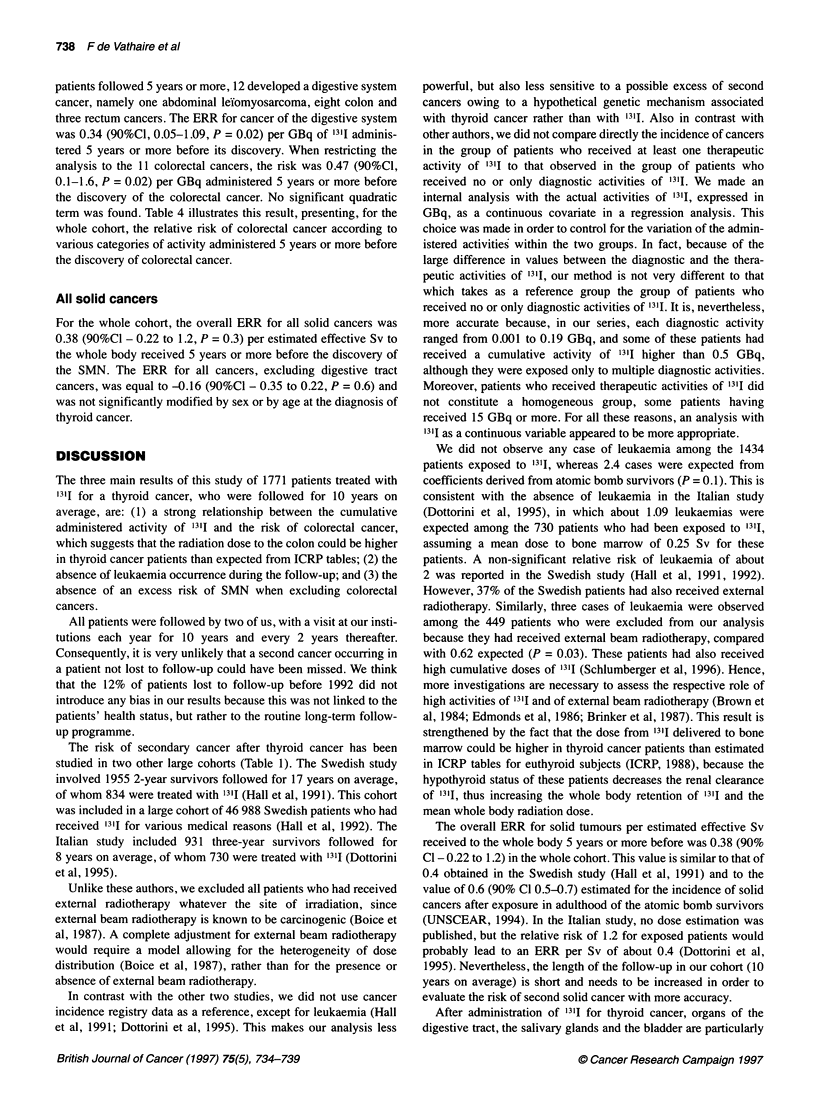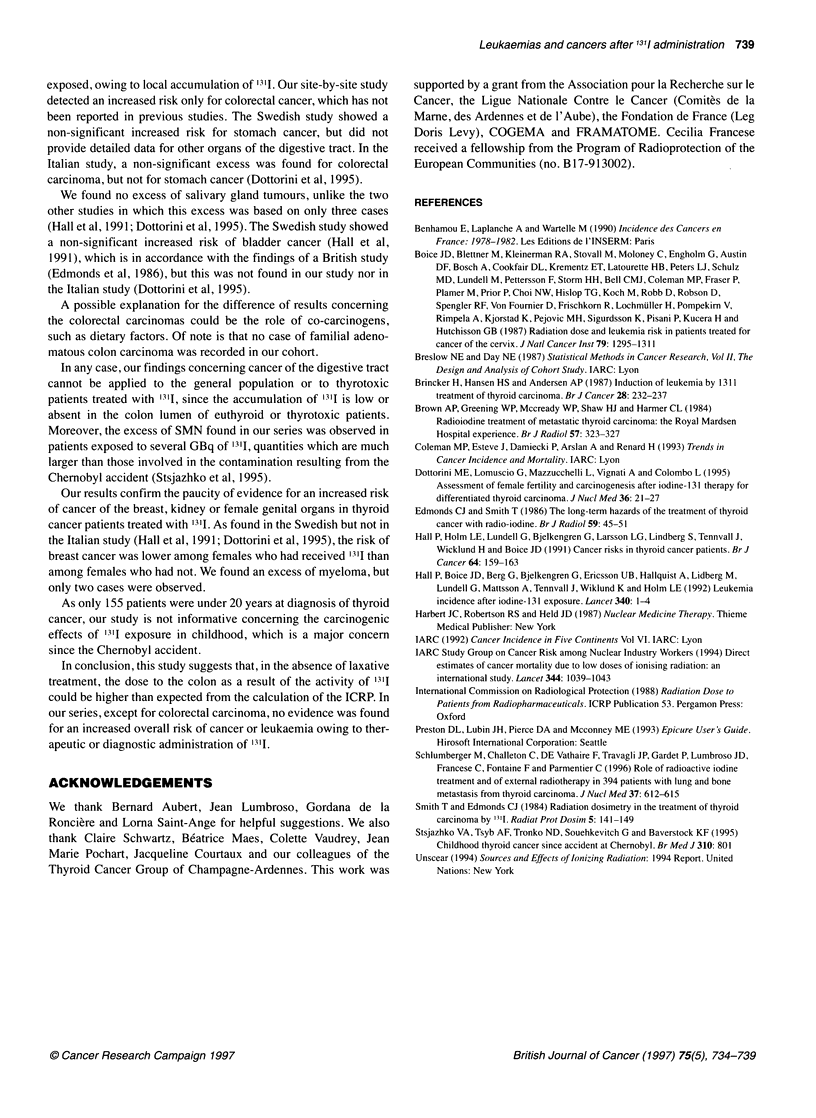# Leukaemias and cancers following iodine-131 administration for thyroid cancer.

**DOI:** 10.1038/bjc.1997.130

**Published:** 1997

**Authors:** F. de Vathaire, M. Schlumberger, M. J. Delisle, C. Francese, C. Challeton, E. de la Genardiére, F. Meunier, C. Parmentier, C. Hill, H. Sancho-Garnier

**Affiliations:** National Institute of Health and of Medical Research, Unit 351, Institut Gustave Roussy, Villejuif, France.

## Abstract

**Images:**


British Journal of Cancer (1997) 75(5), 734–739
© 1997 Cancer Research Campaign

### Leukaemias and cancers following iodine-131 administration for thyroid cancer

**F de Vathaire[1], M Schlumberger[2], MJ Delisle[3], C Francese[1,2], C Challeton[2], E de la Genardiére[1,2], F Meunier[3], C Parmentier[2], C Hill[4] and H Sancho-Garnier[1,4]**

[1]National Institute of Health and of Medical Research, Unit 351, [2]Department of Nuclear Medicine, Institut Gustave Roussy, Rue Camille Desmoulins, 94805 Villejuif Cedex, France; [3]Department of Nuclear Medicine, Institut Jean Godinot, 1 rue du général Koenig, 51056 Reims, France; [4]Department of Biostatistics and Epidemiology, Institut Gustave Roussy, Rue Camille Desmoulins, 94805 Villejuif Cedex, France

**Summary** We studied 1771 patients treated for a thyroid cancer in two institutions. None of these patients had been treated with external radiotherapy and 1497 had received $^{131}$I. The average $^{131}$I cumulative activity administered was 7.2 GBq, and the estimated average dose was 0.34 Sv to the bone marrow and 0.80. Sv to the whole body. After a mean follow-up of 10 years, no case of leukaemia was observed, compared with 2.5 expected according to the coefficients derived from Japanese atomic bomb survivors ($P$ = 0.1). A total of 80 patients developed a solid second malignant neoplasm (SMN), among whom 13 developed a colorectal cancer. The risk of colorectal cancer was found to be related to the total activity of $^{131}$I administered 5 years or more before its diagnosis (excess relative risk = 0.5 per GBq, $P$ = 0,02). These findings were probably caused by the accumulation of $^{131}$I in the colon lumen. Hence, in the absence of laxative treatment, the dose to the colon as a result of $^{131}$I administered for the treatment of thyroid cancer could be higher than expected from calculation of the International Commission on Radiological Protection (ICRP). When digestive tract cancers were excluded, the overall excess relative risk of second cancer per estimated effective sievert received to the whole body was –0.2 ($P$ = 0.6).

**Keywords:** radiocarcinogenesis; carcinogenic effects of $^{131}$I; protracted exposure to radiation; thyroid cancer

On account of the small population dose involved, published studies (IARC, 1994) of nuclear industry workers do not have sufficient power to make reliable comparisons with the risks estimated from the Japanese atomic bomb survivors (UNSCEAR, 1994). Hence, studies of adult populations undergoing protracted exposure to radiation for medical reasons are still necessary. For this purpose, $^{131}$I is a suitable model, because its physical half-life is 8 days. The risks of solid tumours and leukaemia after administration of $^{131}$I have been studied in several cohorts of adults (Hall et al 1991, 1992; Dottorini et al, 1995); nevertheless, the statistical power of these studies is still insufficient to derive precise coefficients.

Another reason for studying the carcinogenic effects of $^{131}$I is the fact that considerable amounts of various radioisotopes of iodine, including $^{131}$I, were released in the atmosphere during the Chernobyl accident. A substantial increase in thyroid cancer incidence has been observed in children living in the most heavily contaminated areas at the time of the accident (Stsjazhko et al. 1995), but no increased incidence of other malignancies, including leukaemia, has been reported so far. More data are thus needed to predict future risks in populations that have been contaminated.

We report the results of a study of 1771 patients treated for a thyroid cancer, of whom 651 had received $^{131}$I for diagnosis, and 846 for therapy.

*Received 5 February 1996*
*Revised 3 September 1996*
*Accepted 20 September 1996*

*Correspondence to*: F de Vathaire

### PATIENTS AND METHODS

### Patients

From 1989 to 1995, all 2479 patients treated for a differentiated thyroid cancer in the two participating institutions between 1950 and December 1989 were studied. The same protocols of treatment and follow-up were used in both centres.

Among the 2479 patients, 173 patients were excluded because they had died or were lost to follow-up during the first two years after the diagnosis of thyroid cancer; 449 patients because they had received external beam radiotherapy, known to be carcinogenic (among whom three developed a leukaemia) and 86 patients because they had had another cancer before thyroid cancer or during the first two years of follow-up.

The remaining 1771 patients were included in the present analysis, of whom 21% were male (Table 1). The mean age at the first treatment of thyroid cancer was 40 years (range 5–89 years). The histology of the thyroid tumour was papillary in 72% of the patients, well-differentiated follicular in 13% and poorly differentiated follicular in 12%. The thyroid tumour was clinically occult in 8% of the patients, was associated with a goitre in 23% and with Graves' disease in 2%.

All patients except 11 underwent thyroid surgery: 64% a total thyroidectomy, 34% a lobo-isthmusectomy, 1% an isthmusectomy and 1% a tumorectomy. Sixty-nine per cent of the patients had lymph node dissection. Post-operatively, a standard ablative dose of 3.7 GBq (100 mCi) was given to patients with poor prognostic indicators or with persistence of neoplastic tissue. After the initial treatment, all patients were followed by two of us (MS and MJD) at a yearly interval for 10 years and then every 2 years. A total body scan with a diagnostic activity of $^{131}$I ranging from 19 to 190

**Table 1** General characteristics of the present cohort and of the other two major cohort studies concerning the effect of $^{131}I$ administered for thyroid cancer

| | Present cohort | Swedish cohort | Italian cohort |
|---|---|---|---|
| Patients | | | |
| Number | 1771 | 1955 | 931 |
| Treatment period | 1950–1989 | 1950–1975 | 1960–1990 |
| Males (%) | 21 | 25 | 25 |
| Mean age at thyroid cancer diagnosis (years) | 40 | 48 | 42 |
| Total thyroidectomy (%) | 64 | 42 | 61 |
| Treatments | | | |
| External beam radiotherapy (%) | 0 | 37 | 6 |
| No $^{131}I$ | 274 (16%) | | |
| Only diagnostic $^{131}I$ administrations | 651 (37%) | 1121 (57%) | 201 (22%) |
| Mean cumulative $^{131}I$ activity in GBq (range) | 0.6 (0.002–10.9) | ? | ? |
| Therapeutic administration of $^{131}I$ | 846 (48%) | 834 (43%) | 730 (78%) |
| Mean cumulative $^{131}I$ activity in GBq (range) | 7.2 (3.8–57.6) | 4.8 (0.48–50.3) | 4.6 (1.9–44.4) |
| Mean 24-h uptake | 13% | 13% | ? |
| Mean dose to bone marrow in Sv (range)[a] | 0.34 (0.13–2.8) | 0.25(0.03–2.2) | ? |
| Follow-up | | | |
| Mean duration in years | 10 | 16 | 8 |
| End of follow-up | 1992 | 1984 | 1993 |
| Second leukaemias | 0 | 8 | 0 |
| Second solid malignant neoplasms | 80 | 213 | 31 |

[a]According to ICRP tables.

MBq (0.5–5 mCi) was performed each year for the first 2 years of follow-up and then every 5 years.

The histological diagnosis of the second primary malignancies was reviewed for 90% of the cases. Cause of death was obtained in 138 of the 143 patients who died during the study.

### Dosimetry

For each diagnostic or therapeutic administration of $^{131}I$, we recorded the day of administration and the activity administered; the sites, the percentage of uptake in the neck, lungs and bones, as shown by $^{131}I$ total body scan (TBS), and the number of days from $^{131}I$ to TBS were also recorded.

A total of 274 patients did not receive any $^{131}I$, 501 were exposed to one or several diagnostic activities of $^{131}I$ equal or less than 0.19 GBq. These patients received a cumulative activity ranging from 0.002 to 0.79 GBq (mean = 0.15 GBq). A total of 292 patients were also exposed to activities of $^{131}I$ higher than 0.19 GBq for diagnostic purposes, but were not given therapeutic activities of 3.7 GBq or more. They received a cumulative activity from 0.20 to 10.9 GBq (mean = 1.2 GBq). The 846 other patients received at least one therapeutic activity of 3.7 GBq or higher, leading to a cumulative activity from 3.8 to 57.6 GBq (mean = 7.2 GBq).

Quantitative uptake in lung and bone as well as the day of measurement were recorded for 5532 (93%) of the 5948 administrations $\leq$ 0.19 GBq, and for 1385 (85%) of the 1629 administrations > 0.19 GBq.

After the administration of 0.19 GBq of $^{131}I$ or less, the average total uptake was 22% at 24 h ($n$ = 395 administrations), 18% at 2 days ($n$ = 925), 7% at 3 days ($n$ = 4076), 3% at 4 days ($n$ = 41) and 5% at 5 days ($n$ = 509). After the administration of more than 0.19 GBq of $^{131}I$, the average total uptake was 13% at 24 h ($n$ = 14 administrations), 13% at 2 days ($n$ = 15), 5% at 3 days ($n$ = 244), 3% at 4 days ($n$ = 306), 8% at 5 days ($n$ = 1027) and 5% at 6 days ($n$ = 23).

In the absence of any other simple method, we added the neck, bone and lung uptake values and entered this 'total' uptake under neck uptake in the International Commission on Radiological Protection (ICRP) tables (ICRP, 1988), in order to estimate the dose delivered to various organs, assuming a biological half-life of 4 days for $^{131}I$ (Harbert et al, 1987). If not available ($n$ = 416), uptake after diagnostic administration was estimated as a linear function of the variables shown to play a role in our cohort: administered activity, age at diagnosis (>/< 40 years), presence of a goitre (yes/no) and extent of thyroid surgery (total/not total). In the same way, if not available, uptake after a therapeutic administration ($n$ = 244) was estimated as a linear function of age at diagnosis (>/< 40 years), extent of thyroid surgery (total/not total) and order of the therapeutic administration (first therapeutic administration or subsequent).

The mean whole body effective dose equivalent was estimated using ICRP tables (ICRP, 1988), assuming a null dose to the thyroid. According to these tables, the 1497 patients exposed to $^{131}I$ received a mean whole body equivalent dose equal to 0.49 Sv, a mean dose to active bone marrow of 0.21 Sv and a mean dose to the breast of 0.18 Sv. In the 846 patients who had received at least one therapeutic activity of 3.7 GBq or more, these doses were 0.80 Sv, 0.34 Sv and 0.29 Sv respectively. The difference between the dose to the whole body and to the active bone marrow resulted from the high doses to organs in which there is an accumulation of $^{131}I$ and which were taken into account in the estimation of the whole body effective dose equivalent: bladder, digestive tract organs and kidney. In our series, a therapeutic administration of 3.7 GBq delivered a mean estimated dose to active bone marrow of 0.16 Sv, and a mean effective dose to the whole body of 0.39 Sv.

ICRP tables were designed for intravenous administrations of radioiodine and may be adapted to oral administration of radioiodine by multiplying by 1.3 the estimations of the dose to the stomach (ICRP, 1988). In these tables, the radiation dose to the upper and lower part of the colon does not vary significantly with

© Cancer Research Campaign 1997

**Figure 1** Example of a total body scan performed 4 days after a therapeutic administration of 3.7 GBq of $^{131}$I. Uptakes are expressed as percentages of the administered activity of $^{131}$I. No laxative treatment was given to this patient. S, stomach; I, intestine; B, bladder

**Figure 2** Cumulative incidence of solid second malignant neoplasms and 95% CI

uptake. In euthyroid subjects, the accumulation of radioiodine in colon is low or absent. In contrast, thyroid cancer patients are hypothyroid at the time of the administration of $^{131}$I, and considerable activities (from 1% to 3% at 4 days) were observed in the colon lumen, due to a decrease in bowel movements. Figure 1 presents a total body scan performed 4 days after the administration of 3.7 GBq of $^{131}$I. As a consequence, laxative treatment has been given after each therapeutic administration since 1985.

Because of this inconsistency with ICRP tables, and as, to our knowledge, no estimation of the dose to colon after $^{131}$I administration to thyroid cancer patients has been published (Smith et al, 1984), we did not perform an estimation of doses to organs of the digestive tract, and we performed the analysis of the risk of colorectal cancer as a function of the cumulative administered activity of $^{131}$I.

### Statistical procedure

The end point of the study was at 1 January 1992. Hence, patients were considered at risk of second cancer during the period beginning 2 years after the diagnosis of their thyroid cancer and ending at the date of the end of follow-up. This date was defined as the first of the four following events: (1) 1st January 1992;(2) occurrence of a second cancer;(3) death of the patient;(4) last visit of the patient to one our institutions. Patients who were not seen on 1 January 1992 or later, and who were alive and without second primary at their last visit were defined as lost to follow-up.

Since there is no national cancer registry in France and since local registries did not cover the study period, the main analysis used internal comparisons. The risk of second malignant neoplasm (SMN) was modelled, using internal analysis, as a function $^{131}$I (in GBq) administered 5 years or more before its occurrence, as continuous covariates. This period of time of 5 years was chosen because radiation delivered less than 5 years before the development of a solid cancer is very unlikely to have contributed to it. This method enabled us to control for the variations of the cumulative administered activities within two groups, i.e. in those who received only diagnostic $^{131}$I and those who were also treated with $^{131}$I. As compared with external analysis, this method also enabled us to control for a hypothetical predisposition of thyroid cancer patients to develop another cancer subsequently, regardless of treatment.

The number of cancers was assumed to follow a Poisson distribution (Breslow et al, 1987). The excess relative risk (ERR) of cancer for a given treatment centre, calendar period of 10 years, sex and attained age was modelled as a linear or/and quadratic function of cumulative activity of $^{131}$I administered 5 years or more before. Modifiers of the effect of $^{131}$I activity were tested. The following models were used:

(1) $ERR = 0$

(2) $ERR = (\gamma_1 b + \gamma_2 b^2) \exp(\alpha_1 a_1 + \alpha_2 a_2)$

as well as intermediate models obtained by selecting any of the following constraints: $\gamma_2 = \alpha_1 = \alpha_2 = 0$, $\gamma_1 = \alpha_1 = \alpha_2 = 0$, $\alpha_2 = 0$ or $\alpha_1 = 0$, where $a_1 = 0$ for male, 1 for female;

$a_2 = 1$ for a patient over age 40 at the time of thyroid cancer treatment, 0 otherwise; $b$ = cumulative activity of $^{131}$I, or estimated dose to organs of interest according to the analysis, delivered 5 years or more before.

The significance of the parameters was tested by comparing the deviances of nested models. The analysis was done using AMFIT software (Preston et al, 1993).

We used external comparisons for leukaemia, because no variation in leukaemia incidence has been observed in Europe over more than 30 years (Coleman et al, 1993; IARC, 1992). We therefore estimated the expected number of leukaemias in our cohort, using incidence rates of leukaemia by age and sex estimated for France for the period 1978–1982 (Benhamou et al, 1990).

### RESULTS

Of the 1771 patients included in the study, 143 died and 220 (12%) were lost to follow-up. The mean follow-up period after the

© Cancer Research Campaign 1997

**Table 2** Characteristics of the 80 second malignant neoplasms (SMNs) observed in the 1771 patients

| Cancer site (ICD9 code) | Patients (*n*) | Years since thyroid cancer | | Age at diagnosis of SMN (years) | | Cumulative $^{131}$I activity before the SMN (GBq) | |
|---|---|---|---|---|---|---|---|
| | | Mean | Min–max | Mean | Min–max | Mean | Min–max |
| Oral cavity (140–149) | 3 | 9 | 4–14 | 49 | 47–51 | 0.1 | 0–0.2 |
| Salivary gland (142) | 1 | 9 | – | 50 | – | 0.2 | – |
| Digestive tract (150–159) | 16 | 13 | 2–29 | 62 | 34–86 | 5.9 | 0.06–22.9 |
| Colon (153) | 10 | 13 | 3–31 | 59 | 34–73 | 3.6 | 0.09–11.7 |
| Rectum (154) | 3 | 19 | 8–29 | 67 | 57–86 | 12.9 | 0.09–22.9 |
| Respiratory organ (160–163) | 4 | 7 | 2–11 | 62 | 49–70 | 4.6 | 0–13.7 |
| Bone (170) | 1 | 8 | – | 26 | – | 6.4 | – |
| Skin melanoma (172) | 4 | 10 | 7–19 | 50 | 37–61 | 0.2 | 0.04–0.4 |
| Other skin tumour (173) | 9 | 14 | 5–32 | 61 | 45–80 | 2.7 | 0–9.8 |
| Breast cancer (174–175) | 21 | 12 | 2–37 | 51 | 30–68 | 0.9 | 0–3.9 |
| Female genital organ (179–184) | 7 | 8 | 2–13 | 63 | 51–82 | 2.4 | 0–8.0 |
| Male genital organ (185–187) | 1 | 18 | – | 86 | – | 0.1 | – |
| Kidney (189) | 2 | 16 | 16–16 | 66 | 54–77 | 0.2 | 0.06–0.4 |
| Nervous system (191–192) | 2 | 8 | 5–11 | 56 | 52–59 | 5.9 | 4.0–7.8 |
| Endocrine gland (194) | 0 | – | – | – | – | – | – |
| Lymphoma (200–202) | 4 | 4 | 2–8 | 52 | 21–65 | 2.4 | 0.5–3.9 |
| Myeloma (203) | 2 | 9 | 7–11 | 61 | 57–64 | 7.7 | 4.0–11.4 |
| Others | 4 | 9 | 2–22 | 51 | 43–62 | 3.0 | 0–7.9 |
| Total | 80 | 11 | 2–37 | 57 | 21–86 | 2.9 | 0–22.9 |

**Table 3** Distribution of the 60 second cancers, which occurred more than 5 years after first cancer diagnosis, according to the type of $^{131}$I administrations ≥ 5 years after treatment

| Cancer site (ICD9 code) | $^{131}$I administration | | |
|---|---|---|---|
| | No $^{131}$I ($n$ = 162) | Only diagnostic activities of $^{131}$I < 3.7 GBq ($n$ = 520) | At least one therapeutic activity of $^{131}$I ≥ 3.7 GBq ($n$ = 593) |
| Oral cavity (140–149) | 1 | 1 | |
| Digestive tract (150–159) | | 5 | 7 |
| Respiratory organ (160–163) | 1 | | 2 |
| Bone (170) | | 1 | |
| Skin melanoma (172) | | 4 | |
| Other skin tumour (173) | 1 | 3 | 5 |
| Breast cancer (174–175) | 4 | 7 | 3 |
| Female genital organ (179–184) | 1 | 3 | 1 |
| Male genital organ (185–187) | | 1 | |
| Kidney (189) | | 2 | |
| Nervous system (191–192) | | 1 | |
| Lymphoma (200–202) | | 2 | |
| Myeloma (203) | | 2 | |
| Others | 1 | | 1 |
| Total | 9 | 27 | 24 |

diagnosis of thyroid cancer was 10 years (range 2–37 years). When the first two years of follow-up were excluded, a total of 14 615 person–years at risk were observed.

Between 2 to 37 years after the diagnosis of thyroid cancer, 80 patients developed a SMN (Figure 2 and Table 2). Of the 1275 patients followed at least 5 years, 60 developed a SMN 5 years or more after first cancer diagnosis (table 3). No difference in the frequency of SMN according to sex, histology or to the presence of a goitre was observed.

**Table 4** Relative risk (RR) of colorectal carcinoma as a function of the cumulative activity of $^{131}$I administered 5 years or more before the diagnosis of colorectal cancer

| Cumulative activity of $^{131}$I (GBq) administered 5 years or more before | Patients (*n*) | Colorectal carcinomas | | |
|---|---|---|---|---|
| | | *n* | Relative risk | 90%CI |
| 0–0.19 | 1051 | 6 | 1.0[a] | |
| > 0.19–3.7 | 184 | 1 | 1.4 | (0.2–6.8) |
| > 3.7–7.5 | 380 | 4 | 4.0 | (1.3–12.2) |
| > 7.5 | 156 | 2 | 4.9 | (1.2–18.5) |

[a]Reference category. Poisson's regression analysis was stratified on sex, treatment centre, calendar period and attained age.

Among the 155 patients aged less than 20 years at the diagnosis of thyroid cancer, 84 received therapeutic activities of $^{131}$I. Five SMNs occurred, two breast carcinomas, one bone sarcoma, one basocellular skin carcinoma and one non-Hodgkin's lymphoma. Only one of these SMNs occurred after a therapeutic administration of $^{131}$I.

### Leukaemias

No case of leukaemia was observed among the 1497 patients exposed to $^{131}$I, compared with 1.28 cases expected from French general population data (Benhamou et al, 1990). Assuming a mean total dose to the bone marrow of 0.21 Sv and using an ERR per Sv for all leukaemia types of 4.4 obtained from the study of adult atomic bomb survivors (UNSCEAR, 1994), 2.5 leukaemias were expected, compared with 0 observed ($P$ = 0.1).

### Digestive tract cancers

Among the whole cohort, 16 patients developed a cancer of the digestive system, of which 13 were colorectal. Among the 1275

© *Cancer Research Campaign 1997*

patients followed 5 years or more, 12 developed a digestive system cancer, namely one abdominal leïomyosarcoma, eight colon and three rectum cancers. The ERR for cancer of the digestive system was 0.34 (90%Cl, 0.05–1.09, $P = 0.02$) per GBq of $^{131}$I administered 5 years or more before its discovery. When restricting the analysis to the 11 colorectal cancers, the risk was 0.47 (90%Cl, 0.1–1.6, $P = 0.02$) per GBq administered 5 years or more before the discovery of the colorectal cancer. No significant quadratic term was found. Table 4 illustrates this result, presenting, for the whole cohort, the relative risk of colorectal cancer according to various categories of activity administered 5 years or more before the discovery of colorectal cancer.

### All solid cancers

For the whole cohort, the overall ERR for all solid cancers was 0.38 (90%Cl – 0.22 to 1.2, $P = 0.3$) per estimated effective Sv to the whole body received 5 years or more before the discovery of the SMN. The ERR for all cancers, excluding digestive tract cancers, was equal to –0.16 (90%Cl – 0.35 to 0.22, $P = 0.6$) and was not significantly modified by sex or by age at the diagnosis of thyroid cancer.

### DISCUSSION

The three main results of this study of 1771 patients treated with $^{131}$I for a thyroid cancer, who were followed for 10 years on average, are: (1) a strong relationship between the cumulative administered activity of $^{131}$I and the risk of colorectal cancer, which suggests that the radiation dose to the colon could be higher in thyroid cancer patients than expected from ICRP tables; (2) the absence of leukaemia occurrence during the follow-up; and (3) the absence of an excess risk of SMN when excluding colorectal cancers.

All patients were followed by two of us, with a visit at our institutions each year for 10 years and every 2 years thereafter. Consequently, it is very unlikely that a second cancer occurring in a patient not lost to follow-up could have been missed. We think that the 12% of patients lost to follow-up before 1992 did not introduce any bias in our results because this was not linked to the patients' health status, but rather to the routine long-term follow-up programme.

The risk of secondary cancer after thyroid cancer has been studied in two other large cohorts (Table 1). The Swedish study involved 1955 2-year survivors followed for 17 years on average, of whom 834 were treated with $^{131}$I (Hall et al, 1991). This cohort was included in a large cohort of 46 988 Swedish patients who had received $^{131}$I for various medical reasons (Hall et al, 1992). The Italian study included 931 three-year survivors followed for 8 years on average, of whom 730 were treated with $^{131}$I (Dottorini et al, 1995).

Unlike these authors, we excluded all patients who had received external radiotherapy whatever the site of irradiation, since external beam radiotherapy is known to be carcinogenic (Boice et al, 1987). A complete adjustment for external beam radiotherapy would require a model allowing for the heterogeneity of dose distribution (Boice et al, 1987), rather than for the presence or absence of external beam radiotherapy.

In contrast with the other two studies, we did not use cancer incidence registry data as a reference, except for leukaemia (Hall et al, 1991; Dottorini et al, 1995). This makes our analysis less powerful, but also less sensitive to a possible excess of second cancers owing to a hypothetical genetic mechanism associated with thyroid cancer rather than with $^{131}$I. Also in contrast with other authors, we did not compare directly the incidence of cancers in the group of patients who received at least one therapeutic activity of $^{131}$I to that observed in the group of patients who received no or only diagnostic activities of $^{131}$I. We made an internal analysis with the actual activities of $^{131}$I, expressed in GBq, as a continuous covariate in a regression analysis. This choice was made in order to control for the variation of the administered activities within the two groups. In fact, because of the large difference in values between the diagnostic and the therapeutic activities of $^{131}$I, our method is not very different to that which takes as a reference group the group of patients who received no or only diagnostic activities of $^{131}$I. It is, nevertheless, more accurate because, in our series, each diagnostic activity ranged from 0.001 to 0.19 GBq, and some of these patients had received a cumulative activity of $^{131}$I higher than 0.5 GBq, although they were exposed only to multiple diagnostic activities. Moreover, patients who received therapeutic activities of $^{131}$I did not constitute a homogeneous group, some patients having received 15 GBq or more. For all these reasons, an analysis with $^{131}$I as a continuous variable appeared to be more appropriate.

We did not observe any case of leukaemia among the 1434 patients exposed to $^{131}$I, whereas 2.4 cases were expected from coefficients derived from atomic bomb survivors ($P = 0.1$). This is consistent with the absence of leukaemia in the Italian study (Dottorini et al, 1995), in which about 1.09 leukaemias were expected among the 730 patients who had been exposed to $^{131}$I, assuming a mean dose to bone marrow of 0.25 Sv for these patients. A non-significant relative risk of leukaemia of about 2 was reported in the Swedish study (Hall et al, 1991, 1992). However, 37% of the Swedish patients had also received external radiotherapy. Similarly, three cases of leukaemia were observed among the 449 patients who were excluded from our analysis because they had received external beam radiotherapy, compared with 0.62 expected ($P = 0.03$). These patients had also received high cumulative doses of $^{131}$I (Schlumberger et al, 1996). Hence, more investigations are necessary to assess the respective role of high activities of $^{131}$I and of external beam radiotherapy (Brown et al, 1984; Edmonds et al, 1986; Brinker et al, 1987). This result is strengthened by the fact that the dose from $^{131}$I delivered to bone marrow could be higher in thyroid cancer patients than estimated in ICRP tables for euthyroid subjects (ICRP, 1988), because the hypothyroid status of these patients decreases the renal clearance of $^{131}$I, thus increasing the whole body retention of $^{131}$I and the mean whole body radiation dose.

The overall ERR for solid tumours per estimated effective Sv received to the whole body 5 years or more before was 0.38 (90% Cl – 0.22 to 1.2) in the whole cohort. This value is similar to that of 0.4 obtained in the Swedish study (Hall et al, 1991) and to the value of 0.6 (90% Cl 0.5–0.7) estimated for the incidence of solid cancers after exposure in adulthood of the atomic bomb survivors (UNSCEAR, 1994). In the Italian study, no dose estimation was published, but the relative risk of 1.2 for exposed patients would probably lead to an ERR per Sv of about 0.4 (Dottorini et al, 1995). Nevertheless, the length of the follow-up in our cohort (10 years on average) is short and needs to be increased in order to evaluate the risk of second solid cancer with more accuracy.

After administration of $^{131}$I for thyroid cancer, organs of the digestive tract, the salivary glands and the bladder are particularly

© Cancer Research Campaign 1997

exposed, owing to local accumulation of $^{131}$I. Our site-by-site study detected an increased risk only for colorectal cancer, which has not been reported in previous studies. The Swedish study showed a non-significant increased risk for stomach cancer, but did not provide detailed data for other organs of the digestive tract. In the Italian study, a non-significant excess was found for colorectal carcinoma, but not for stomach cancer (Dottorini et al, 1995).

We found no excess of salivary gland tumours, unlike the two other studies in which this excess was based on only three cases (Hall et al, 1991; Dottorini et al, 1995). The Swedish study showed a non-significant increased risk of bladder cancer (Hall et al, 1991), which is in accordance with the findings of a British study (Edmonds et al, 1986), but this was not found in our study nor in the Italian study (Dottorini et al, 1995).

A possible explanation for the difference of results concerning the colorectal carcinomas could be the role of co-carcinogens, such as dietary factors. Of note is that no case of familial adenomatous colon carcinoma was recorded in our cohort.

In any case, our findings concerning cancer of the digestive tract cannot be applied to the general population or to thyrotoxic patients treated with $^{131}$I, since the accumulation of $^{131}$I is low or absent in the colon lumen of euthyroid or thyrotoxic patients. Moreover, the excess of SMN found in our series was observed in patients exposed to several GBq of $^{131}$I, quantities which are much larger than those involved in the contamination resulting from the Chernobyl accident (Stsjazhko et al, 1995).

Our results confirm the paucity of evidence for an increased risk of cancer of the breast, kidney or female genital organs in thyroid cancer patients treated with $^{131}$I. As found in the Swedish but not in the Italian study (Hall et al, 1991; Dottorini et al, 1995), the risk of breast cancer was lower among females who had received $^{131}$I than among females who had not. We found an excess of myeloma, but only two cases were observed.

As only 155 patients were under 20 years at diagnosis of thyroid cancer, our study is not informative concerning the carcinogenic effects of $^{131}$I exposure in childhood, which is a major concern since the Chernobyl accident.

In conclusion, this study suggests that, in the absence of laxative treatment, the dose to the colon as a result of the activity of $^{131}$I could be higher than expected from the calculation of the ICRP. In our series, except for colorectal carcinoma, no evidence was found for an increased overall risk of cancer or leukaemia owing to therapeutic or diagnostic administration of $^{131}$I.

### ACKNOWLEDGEMENTS

We thank Bernard Aubert, Jean Lumbroso, Gordana de la Roncière and Lorna Saint-Ange for helpful suggestions. We also thank Claire Schwartz, Béatrice Maes, Colette Vaudrey, Jean Marie Pochart, Jacqueline Courtaux and our colleagues of the Thyroid Cancer Group of Champagne-Ardennes. This work was supported by a grant from the Association pour la Recherche sur le Cancer, the Ligue Nationale Contre le Cancer (Comitès de la Marne, des Ardennes et de l'Aube), the Fondation de France (Leg Doris Levy), COGEMA and FRAMATOME. Cecilia Francese received a fellowship from the Program of Radioprotection of the European Communities (no. B17-913002).

### REFERENCES

Benhamou E, Laplanche A and Wartelle M (1990) *Incidence des Cancers en France: 1978–1982*. Les Editions de l'INSERM: Paris

Boice JD, Blettner M, Kleinerman RA, Stovall M, Moloney C, Engholm G, Austin DF, Bosch A, Cookfair DL, Krementz ET, Latourette HB, Peters LJ, Schulz MD, Lundell M, Pettersson F, Storm HH, Bell CMJ, Coleman MP, Fraser P, Plamer M, Prior P, Choi NW, Hislop TG, Koch M, Robb D, Robson D, Spengler RF, Von Fournier D, Frischkorn R, Lochmüller H, Pompekirn V, Rimpela A, Kjorstad K, Pejovic MH, Sigurdsson K, Pisani P, Kucera H and Hutchisson GB (1987) Radiation dose and leukemia risk in patients treated for cancer of the cervix. *J Natl Cancer Inst* **79**: 1295–1311

Breslow NE and Day NE (1987) *Statistical Methods in Cancer Research, Vol II, The Design and Analysis of Cohort Study*. IARC: Lyon

Brincker H, Hansen HS and Andersen AP (1987) Induction of leukemia by 1311 treatment of thyroid carcinoma. *Br J Cancer* **28**: 232–237

Brown AP, Greening WP, Mccready WP, Shaw HJ and Harmer CL (1984) Radioiodine treatment of metastatic thyroid carcinoma: the Royal Mardsen Hospital experience. *Br J Radiol* **57**: 323–327

Coleman MP, Esteve J, Damiecki P, Arslan A and Renard H (1993) *Trends in Cancer Incidence and Mortality*. IARC: Lyon

Dottorini ME, Lomuscio G, Mazzucchelli L, Vignati A and Colombo L (1995) Assessment of female fertility and carcinogenesis after iodine-131 therapy for differentiated thyroid carcinoma. *J Nucl Med* **36**: 21–27

Edmonds CJ and Smith T (1986) The long-term hazards of the treatment of thyroid cancer with radio-iodine. *Br J Radiol* **59**: 45–51

Hall P, Holm LE, Lundell G, Bjelkengren G, Larsson LG, Lindberg S, Tennvall J, Wicklund H and Boice JD (1991) Cancer risks in thyroid cancer patients. *Br J Cancer* **64**: 159–163

Hall P, Boice JD, Berg G, Bjelkengren G, Ericsson UB, Hallquist A, Lidberg M, Lundell G, Mattsson A, Tennvall J, Wiklund K and Holm LE (1992) Leukemia incidence after iodine-131 exposure. *Lancet* **340**: 1–4

Harbert JC, Robertson RS and Held JD (1987) *Nuclear Medicine Therapy*. Thieme Medical Publisher: New York

IARC (1992) *Cancer Incidence in Five Continents* Vol VI. IARC: Lyon

IARC Study Group on Cancer Risk among Nuclear Industry Workers (1994) Direct estimates of cancer mortality due to low doses of ionising radiation: an international study. *Lancet* **344**: 1039–1043

International Commission on Radiological Protection (1988) *Radiation Dose to Patients from Radiopharmaceuticals*. ICRP Publication 53. Pergamon Press: Oxford

Preston DL, Lubin JH, Pierce DA and Mcconney ME (1993) *Epicure User's Guide*. Hirosoft International Corporation: Seattle

Schlumberger M, Challeton C, DE Vathaire F, Travagli JP, Gardet P, Lumbroso JD, Francese C, Fontaine F and Parmentier C (1996) Role of radioactive iodine treatment and of external radiotherapy in 394 patients with lung and bone metastasis from thyroid carcinoma. *J Nucl Med* **37**: 612–615

Smith T and Edmonds CJ (1984) Radiation dosimetry in the treatment of thyroid carcinoma by $^{131}$I. *Radiat Prot Dosim* **5**: 141–149

Stsjazhko VA, Tsyb AF, Tronko ND, Souehkevitch G and Baverstock KF (1995) Childhood thyroid cancer since accident at Chernobyl. *Br Med J* **310**: 801

Unscear (1994) *Sources and Effects of Ionizing Radiation*: 1994 Report. United Nations: New York

© Cancer Research Campaign 1997